# Improving Social Relationships in Long-Term Care Settings: Insights for Innovative Strategies

**DOI:** 10.1093/geroni/igaf122.871

**Published:** 2025-12-31

**Authors:** E-Shien Chang, Amy Roberts, Noah Webster

## Abstract

Residents of long-term care (LTC) homes represent a unique population with significant needs for meaningful social connections. Building strong social connections with other residents and staff can assist residents in managing the challenges of communal living. In contrast, poor social interactions can seriously affect residents’ quality of care and well-being. Despite the implications of vital social relationships for LTC residents, there has been less focus on understanding the full spectrum of social interactions, both from a resident-resident and resident-staff perspective, to guide intervention programming. In this symposium, we will discuss new insights for building meaningful social relationships across various LTC residential settings. Dr. Amy Roberts will describe existing practices, gaps, and training opportunities for improving social engagement and managing resident conflicts from the viewpoints of social services workers in U.S. nursing homes. Dr. E-Shien Chang will discuss findings from a qualitative study of nursing home clinical and non-clinical providers regarding predisposing factors and acute precipitants for race/ethnicity-related resident-to-resident aggression. Using a social network analysis approach, Dr. Rebecca Mauldin will explore negative social interactions found in social networks among residents of assisted living communities with data from a longitudinal survey. Dr. Elsie Yan will discuss the prevalence and risk factors of resident-initiated aggression towards staff and their impact on resident relationships based on a cohort study conducted in Hong Kong. Finally, Dr. Noah Webster, a policy and intervention expert in the area of social relations, will provide practice recommendations for prioritizing psycho-social needs and relation-based support tailored towards the LTC community.

